# Probenecid-Blocked Pannexin-1 Channel Protects Against Early Brain Injury *via* Inhibiting Neuronal AIM2 Inflammasome Activation After Subarachnoid Hemorrhage

**DOI:** 10.3389/fneur.2022.854671

**Published:** 2022-03-23

**Authors:** Yonghe Zheng, Wenwen Tang, Hanhai Zeng, Yucong Peng, Xiaobo Yu, Feng Yan, Shenglong Cao

**Affiliations:** ^1^Department of Neurosurgery, The Second Affiliated Hospital of Zhejiang University School of Medicine, Hangzhou, China; ^2^Zhejiang University School of Medicine, Hangzhou, China

**Keywords:** AIM2 inflammasome, early brain injury, neurons, pannexin-1, subarachnoid hemorrhage, probenecid

## Abstract

**Aim:**

Previous studies have proved that inhibiting inflammasome activation provides neuroprotection against early brain injury (EBI) after subarachnoid hemorrhage (SAH), which is mainly focused on the microglial inflammatory response, but the potential role of neuronal inflammasome activation in EBI has not been clearly identified. This study examined whether the pannexin-1 channel inhibitor probenecid could reduce EBI after SAH by inhibiting neuronal AIM2 inflammasome activation.

**Methods:**

There are *in vivo* and *in vitro* parts in this study. First, adult male SD rats were subjected to the endovascular perforation mode of SAH. The time course of pannexin-1 and AIM2 expressions were determined after SAH in 72 h. Brain water content, neurological function, AIM2 inflammasome activation, and inflammatory response were evaluated at 24 h after SAH in sham, SAH, and SAH + probenecid groups. In the *in vitro* part, HT22 cell treated with hemin was applied to mimic SAH. The expression of AIM2 inflammasome was detected by immunofluorescence staining. Neuronal death and mitochondrial dysfunction were determined by the LDH assay kit and JC-1 staining.

**Results:**

The pannexin-1 and AIM2 protein levels were upregulated after SAH. Pannexin-1 channel inhibitor probenecid attenuated brain edema and improved neurological dysfunction by reducing AIM2 inflammasome activation and reactive oxygen species (ROS) generation after SAH in rats. Treating HT22 cells with hemin for 12 h resulted in AIM2 and caspase-1 upregulation and increased mitochondrial dysfunction and neuronal cell death. Probenecid significantly attenuated the hemin-induced AIM2 inflammasome activation and neuronal death.

**Conclusions:**

AIM2 inflammasome is activated in neurons after SAH. Pharmacological inhibition of the pannexin-1 channel by probenecid attenuated SAH-induced AIM2 inflammasome activation and EBI *in vivo* and hemin-induced AIM2 inflammasome activation and neuronal death *in vitro*.

## Introduction

Subarachnoid hemorrhage (SAH), which mostly occurs following aneurysm rupture, is a devastating subtype of stroke with high mortality. Several studies have shown that early brain injury (EBI) is a key factor in the poor prognosis of patients with SAH (1, 2). Increased evidence demonstrated that inflammasome-mediated neuroinflammation and neuronal cell death contribute to injury progression in the early stage of SAH (3, 4). Neuronal pyroptosis is defined as a highly specific inflammatory programmed cell death, which is triggered by inflammasome activation (5). Many glial inflammasomes have been identified after stroke, and the most widely studied inflammasome is the NOD-like receptor family pyrin domain containing 3 (NLRP3), which is mainly released by microglia after SAH. Inhibition of NLPR3 inflammasome activation was proved to attenuate EBI after SAH (3, 4, 6). However, only a few studies were focused on the role of neuronal inflammasome-mediated neuroinflammation and pyroptotic neuronal cell death after stroke.

Absent in melanoma 2 (AIM2) is a member of the hemopoietic IFN-inducible nuclear 200 (HIN-200) family of proteins that induce the formation of a highly specific inflammasome in neurons. AIM2 inflammasome could be activated by host ectopic double-stranded DNA (dsDNA) in cortical neurons (7). AIM2 triggers the formation of the inflammasome, which contains apoptosis-associated speck-like proteins containing CARD (ASC) and pro-caspase-1, and induces caspase-1 cleavage, interleukin-1β (IL-1β) and interleukin-18 (IL-18) maturation, and pyroptosis (7, 8). AIM2 inflammasome-mediated pyroptotic neuronal death has been validated in neurodevelopment and central nervous system (CNS) diseases, which include neurodegenerative diseases, hemorrhagic stroke, and traumatic brain injury (8–10). However, the potential therapeutic targets for AIM2 inflammasome activation in the pathogenesis of EBI after SAH have not been clearly clarified.

Pannexin-1 is a new member of the gap junction family of proteins, with homology to invertebrate innexins. Pannexin-1 is highly expressed in many organs, which include the brain, the spinal cord, and bones (11). The pannexin-1 channel carries signaling molecules and ions between the cytoplasm and the extracellular space. As a membrane channel, pannexin-1 is involved in ATP release to extracellular space and the propagation of intercellular calcium waves (12). Emerging evidence reveals the pathological activity of pannexin-1 in contributing to disease processes that include ischemic stroke, seizure, spinal cord injury, and tumor formation (11, 13–15). Moreover, the pannexin-1 channel has been shown to activate the inflammasome of neurons and astrocytes (16). The regulatory role of the pannexin-1 channel on AIM2 inflammasome activation after SAH is rarely known. Probenecid is an “old drug” that has been used as a uricosuric drug for gout treatment, and its pharmacokinetics and side effects have been widely studied (17, 18). Probenecid was found to be a potent-specific inhibitor of pannexin-1 channel (18).

Therefore, this study was designed to evaluate the effect of probenecid-blocked pannexin-1 channel on EBI after SAH and to explore the relationship with AIM2 inflammasome activation. In this study, we found that pannexin-1 channel inhibitor probenecid could inhibit AIM2 inflammasome activation in the acute stage of SAH, could consequently reduce brain edema, and could improve neurological dysfunction.

## Materials and Methods

### Animal Preparation and Study Design

Adult male Sprague-Dawley rats (300–340 g) which were obtained from SLAC Laboratory Co., Ltd. (Shanghai, China) were used in this study. A total of 136 rats were used for this study. The Sprague-Dawley rats were raised in plastic cages with controlled temperature and humidity and a 12-hlight–dark cycle. All animal experiments were in accordance with the Guidelines for the Care and Use of Laboratory Animals of the National Institutes of Health and were approved by the Institutional Animal Care and Use Committee of Zhejiang University.

**Figure 1:**
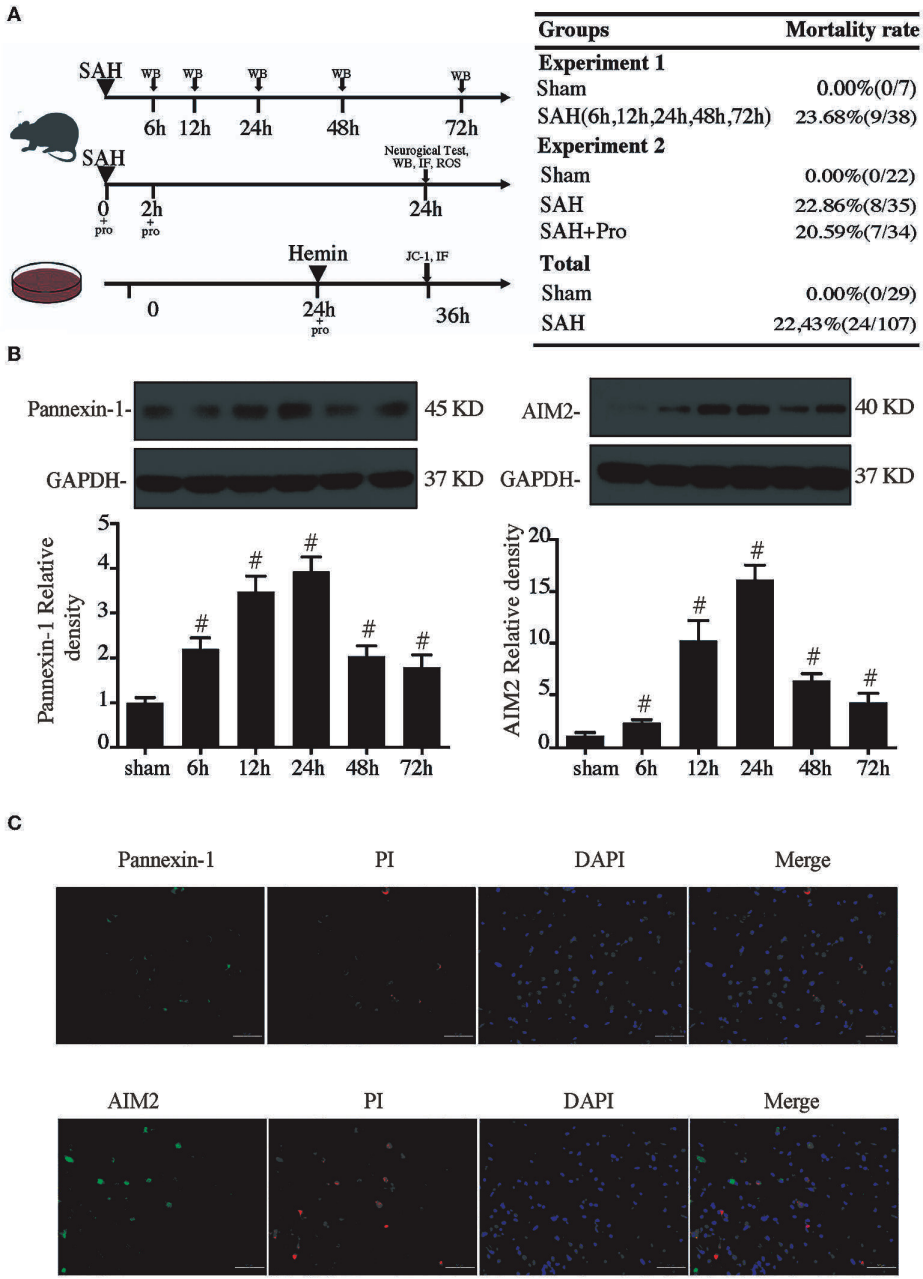
**(A)** Experimental design for *in vivo* and *in vitro* experiments, animal groups, and mortality. **(B)** Time course of pannexin-1 and AIM2 levels, *n* = 4 for each group. #*p* < 0.05 vs. sham group. **(C)** Representative micrographs showing double immunofluorescence labeling of propidium iodide (PI) with pannexin-1 and AIM2 in the ipsilateral basal cortex at 24 h after subarachnoid hemorrhage (SAH). Scale bar = 50 μm.

The *in vivo* study was divided into 2 parts. In the first part, rats were subjected to SAH with an endovascular perforation technique. Brains were harvested at 6, 12, 24, 48, and 72 h after SAH (*n* = 4 each group) for western blots and brain histology (*n* = 4 at 24 h after SAH). Brains of the sham group were harvested at 24 h after surgical procedures. In the second part, SAH groups were subjected to SAH and treated with vehicle or probenecid, and the sham group was subjected to a procedure similar to the SAH groups but without perforation and treatement with vehicle. All endpoints in this part were investigated 24 h after SAH based on the result of the first part. The experimental group design is shown in Figure 1A.

### The Rat SAH Model

The rat SAH model was performed by the endovascular perforation technique as previously described (19). Rats were anesthetized with pentobarbital (40 mg/kg, intraperitoneal injection) to expose the left carotid artery and its branches. The external carotid artery was transected distally, and a blunt 4-0 nylon monofilament suture was inserted into the internal carotid artery, which was advanced through until resistance was felt and occurred at the junction of the anterior cerebral artery and the middle cerebral artery. The suture was further advanced ~3–5 mm to perforate the artery and create SAH with a stay of 10 s. The sham group underwent a similar procedure without perforation.

The SAH grades were quantified in accordance with the published grading system of Zhang et al. (20). The grade scores were based on the volume of a clot in six parts of the basal cistern. Each segment was scored 0–3, and the final scores were obtained by adding six partial scores.

### Cell Culture

The mouse hippocampal neuronal cell line HT22 cells were cultured (37°C, 5% CO_2_) in DMEM/F12 (1:1) medium with 15% fetal bovine serum (FBS) and 100 U/ml penicillin. These cells were inoculated in 5 cm^2^ × 5 cm^2^-cell culture flask at a density range of 4 × 10^6^/well−5 × 10^6^/well. Following SAH, the erythrocytes lyse and release their hemoglobin, which degrades to hemin. The toxicity of hemin occurs after it has been taken up into cells and thereby causes various harmful responses. We stimulated HT22 cells by administrating 75 μm hemin (Sigma-Aldrich, MO, USA, catalog number H9039) for 12 h to induce the SAH model *in vitro*. Meanwhile, cells were treated with probenecid (1 mM probenecid) or vehicles (Figure 1A).

### Drug Administration

*In vivo*: 50 mg probenecid (P8761, Sigma, USA) was dissolved in 2 mM NaOH PH was titrate to 7.0 with 0.2M HCl. Then, water was added to a final volume of 50 ml (1 mg/ml probenecid). Rats were intraperitoneally injected with probenecid at a dose of 1 mg/kg using two-injection protocol as described previously (21, 22). The injection of probenecid or vehicle was performed before SAH and again 2 h after SAH, for a total of two doses.

*In vitro*: HT22 cells were cultured in DMEM/F12 medium for 24 h. Cells were stimulated by hemin, hemin + probenecid, or probenecid for an additional 12 h. The control cells received the same volumes of vehicles.

### Evaluation of Neurological Deficits

The neurological deficits were evaluated 24 h after SAH according to the scoring system of Garcia et al. (23). The scoring system consisted of the following six tests: symmetry in the movement of four limbs, spontaneous activity, climbing, body proprioception, forepaw outstretching, and response to vibrissae touch. Each test was scored 0–3 or 1–3, and the total score ranged from 3 to 18. A lower score represents more serious neurological deficits. All the tests were assessed in a blind manner in a random sequence.

### Brain Water Content Measurement

Rats were sacrificed 24 h after SAH. The brain was removed and immediately weighed to obtain the wet weight (WW). The samples were dried at 105°C for 24 h and weighed again to obtain the dry weight (DW). The brain water content was calculated as [(WW–DW)/WW] × 100% (24).

### ROS Assay

A reactive oxygen species (ROS) assay was performed as previously described with the ROS assay kit (Nanjing Jiancheng Bioengineering Institute, Nanjing, China) following the manufacturer's instruction (25). The ROS levels in the brain tissue were expressed as fluorescence intensity/mg protein.

### Western Blot Analysis

Ipsilateral basal cortical samples facing blood clots were extracted. Western blotting was performed as described previously (25). Cortical samples were homogenized and centrifuged (1,000 × g, 10 min, 4°C). The supernatant was further centrifuged, and then, the protein concentration was determined using the DC protein assay kit (Bio-Rad, Hercules, CA, USA). An equal amount of protein (50 μg) was suspended in loading buffer, denatured at 95°C for 5 min, and loaded on an SDS-PAGE gel. After being electrophoresed and transferred into polyvinylidene fluoride membranes, the membrane was blocked with nonfat dry milk buffer for 2 h and then incubated overnight at 4°C with the primary antibody for AIM2 (ab93015, 1:1,000, Abcam), pannexin-1 (ab124131, 1:1,000, Abcam), ASC (ab155970, 1:1,000, Abcam), caspase-1 (ab1872, 1:1,000, Abcam), P2X7R (1: 1,000, APR-004, Alomone, Jerusalem), IL-1β (SC-23460, 1:500; Santa Cruz), IL-18 (ab71495, 1:1,000; Abcam), and GAPDH (1:10,000, Fitzgerald, 10R-G109A). The membranes were incubated with horseradish peroxidase-conjugated secondary antibodies for 1 h at room temperature. The protein band densities were detected by x-ray film and quantified by ImageJ software (National Institutes of Health, Bethesda, MD, USA).

### Immunofluorescence Staining and JC-1 Staining

Propidium iodide (PI, 25535-16-4, sigma) was dissolved in 0.9% NaCl, and 10 mg/kg PI was administered intraperitoneally at 1 h prior to sacrifice. Brain coronal slices were obtained according to our previous protocol (4). Immunofluorescence staining was performed as previously described. The primary antibodies were AIM2 (ab93015, 1:1,000, Abcam), pannexin-1 (ab124131, 1:1,000, Abcam), and caspase-1 (ab1872, 1:1,000, Abcam). The secondary antibodies were rhodamine-conjugated antibody (1:200, Jackson Immuno Research) and fluorescein isothiocyanate-labeled antibody (1:200, Jackson Immuno Research). The sections were rinsed and stained with DAPI (1 μg/mL, Roche Inc, Basel, Switzerland) and then rinsed again and mounted with glycerol. The labeling was analyzed using a fluorescence microscope (Olympus).

The HT22 cells were inoculated in 96-well plates at a density range of 1 × 10^4^/well−3 × 10^4^/well, and then, these cells were used for immunofluorescence staining and measuring mitochondrial membrane potential. The cultures were washed two times with PBS and fixed with 4% paraformaldehyde in PBS for 15 min and then washed two times more with PBS at room temperature. The staining protocol and the primary and second antibodies were the same as described above. The mitochondrial membrane potential was measured by a JC-1 kit (Beyotime, Shanghai, China) following the manufacturer's instructions. HT22 cells were rinsed with PBS and incubated with JC-1 staining solution at 37°C for 20 min. Then, the inverted fluorescence microscope (Olympus, Tokyo, Japan) was used to capture the pictures and calculate the ratio of the red–green fluorescence.

### LDH Assay

In brief, the HT22 cells were inoculated in 96-well plates at a density range of 1 × 10^4^/well−3 × 10^4^/well. Culture medium (20 μl) was taken after 12 h of treatment, and cell death was quantified by lactate dehydrogenase release assay (Nanjing Jiancheng Bioengineering Institute, Nanjing, China) following the manufacturer's instruction. The LDH release was determined by measuring the 450 nm absorbance using a microplate reader.

### Statistical Analysis

Continuous data were presented as mean ± standard error of the mean (SEM) or median (interquartile range) based on the normality and homogeneity of variance. For the data meeting normal distribution and homogeneity of variance, one-way analysis of variance (ANOVA) followed by a Tukey's multiple comparison test was used to evaluate significant differences among groups. For the non-normal distribution and unequal variance parameters, the Kruskal–Wallis test was used to compare the differences among the groups. The Chi-square test was used for mortality comparison. The *p*-value <0.05 indicated statistical significance. GraphPad Prism (version 9.0) and SPSS software (version 23.0) were used for statistical analyses.

## Results

### The Expressions of Pannexin-1 and AIM2 Were Elevated After SAH

Experimental SAH surgeries were performed in rats with the endovascular perforation technique. The pannexin-1 channel is the principal conduits of ATP release from dying cells in the brain, which release cytoplasmic contents that include dsDNA. AIM2 is a member of the HIN-200 family of proteins and was activated to form an inflammasome in the neuron by dsDNA. Western blot results showed that the pannexin-1 and AIM2 protein contents gradually increased with a marked accumulation at 24 h after SAH and still stay at a high level at 72 h post-SAH when compared to the sham group (*p* < 0.05, Figure 1B). PI staining was used for the detection of necrotic cells. Double immunostaining demonstrated that pannexin-1-positive and AIM2-positive cells were mainly expressed on PI-positive cells after SAH induction (Figure 1C).

### Probenecid-Blocked Pannexin-1 Channel Reduced Brain Water Content and Improved Neurological Dysfunction

**Figure 2:**
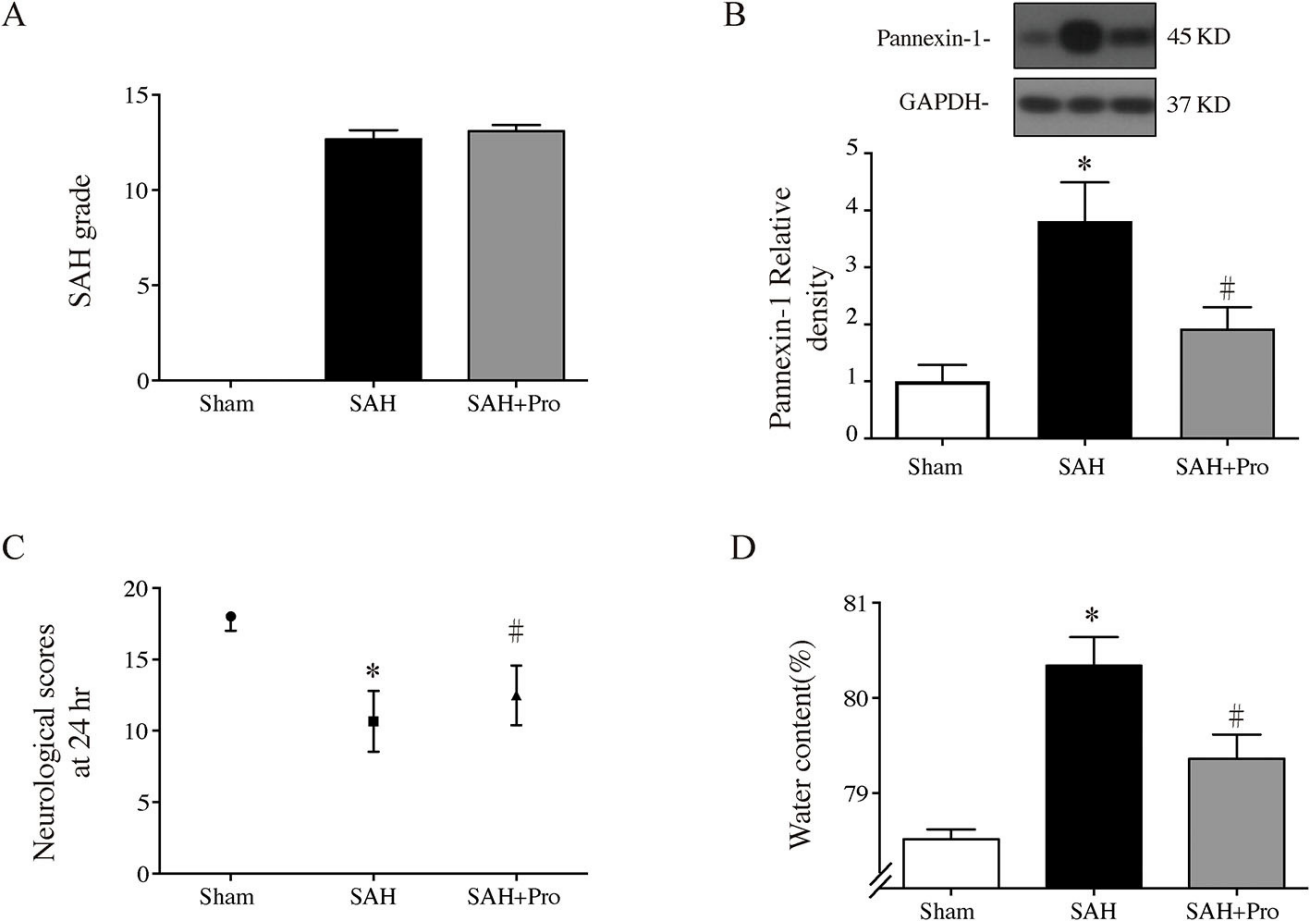
**(A)** The quantification of SAH severity, *n* = 21 for each group. **(B)** Pannexin-1 protein levels in sham, SAH, and SAH + pro groups, *n* = 6 each group. *p < 0.05 vs. sham group, #*p* < 0.05 vs. SAH group. **(C)** The quantification of neurological scores. Values are median (interquartile range), *n* = 21 each group, *p < 0.05 vs. sham group, #*p* < 0.05 vs. SAH group. **(D)** Brain water content measured by the wet–dry method, *n* = 6 each group. *p < 0.05 vs. sham group, #*p* < 0.05 vs. SAH group.

The SAH grade of the sham group was 0, and the scores were not significantly different between SAH groups (Figure 2A). Probenecid is a pharmacological inhibitor of the pannexin-1 channel. Western blot showed that the treatment of probenecid reversed the increase of pannexin-1 protein expression after SAH (*p* < 0.05, Figure 2B). Brain water content was measured by wet–dry method and analyzed 24 h after SAH. The whole-brain water content of the SAH group was significantly higher than that of the sham group. The rats treated with probenecid showed reduced brain edema compared with the SAH group (*p* < 0.05, Figure 2C). The neurological score of the SAH group was significantly lower than that of the sham group. Probenecid treatment improved the neurological scores significantly compared with the SAH group (*n* = 21, *p* < 0.05, Figure 2D). When the mortality of SAH rats was estimated, we found that mortality was slightly lower in the SAH + pro group (20.59%) than in the SAH group (22.86%) with no statistical difference (Figure 1A). These results indicated that probenecid treatment provided a neuroprotective effect on EBI after SAH.

### Probenecid Inhibited AIM2 Inflammasome Activation After SAH

**Figure 3:**
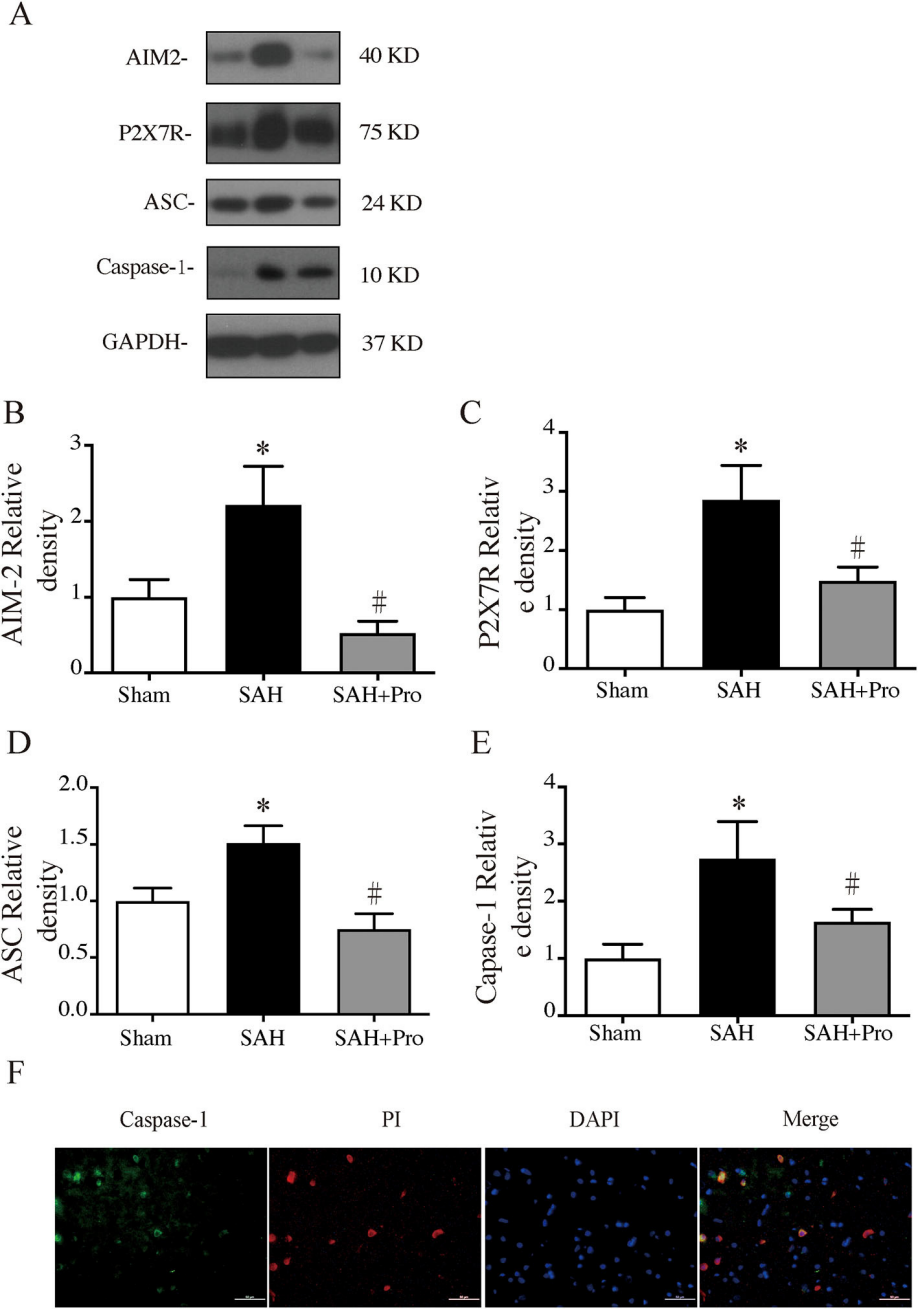
**(A)** Representative western blotting images of AIM2, ATP-activated P2X7 purinergic receptor (P2X7R), apoptosis-associated speck-like proteins containing CARD (ASC), and caspase-1. **(B)** AIM2 protein levels in sham, SAH, and SAH + pro groups, *n* = 6 for each group. *p < 0.05 vs. sham group, #*p* < 0.05 vs. SAH group. **(C)** P2X7R protein levels in sham, SAH, and SAH + pro groups, *n* = 6 for each group. *p < 0.05 vs. sham group, #*p* < 0.05 vs. SAH group. **(D)** ASC protein levels in sham, SAHs and SAH + pro groups, *n* = 6 for each group. *p < 0.05 vs. sham group, #*p* < 0.05 vs. SAH group. **(E)** Caspase-1 protein levels in sham, SAH, and SAH + pro groups, *n* = 6 for each group. *p < 0.05 vs. sham group, #*p* < 0.05 vs. SAH group. **(F)** Representative micrographs showing double immunofluorescence labeling of caspase-1 with PI in the ipsilateral basal cortex at 24 h after SAH. Scale bar = 50 μm.

To assess the AIM2 inflammasome activation after SAH, we measured the expression levels of AIM2, ASC, and caspase-1. Compared with the sham group, the expression levels of AIM2, ASC, and caspase-1 were significantly increased in SAH group. The pannexin-1 inhibitor, probenecid, markedly reduced the protein expressions of AIM2, ASC, and caspase-1(*p* < 0.05, Figures 3A,B,D,E). Double immunostaining showed that caspase-1-positive cells were mainly expressed in PI-positive cells after SAH (Figure 3F). Pannexin-1 channel is associated with the ATP release of dying cells for large pore formation on the cell membrane, and this function is closely tied to that of ATP-activated P2X7 purinergic receptor (P2X7R) (12). Western blot analysis showed that the P2X7R level was significantly elevated 24 h after SAH, and probenecid treatment markedly reduced the expression of P2X7R compared with vehicle-treated rats (*p* < 0.05, Figures 3A,C), which might be due to the inhibition of the pannexin-1 channel.

### Probenecid Reduced Pro-inflammatory Cytokine Secretion and ROS Generation After SAH

**Figure 4:**
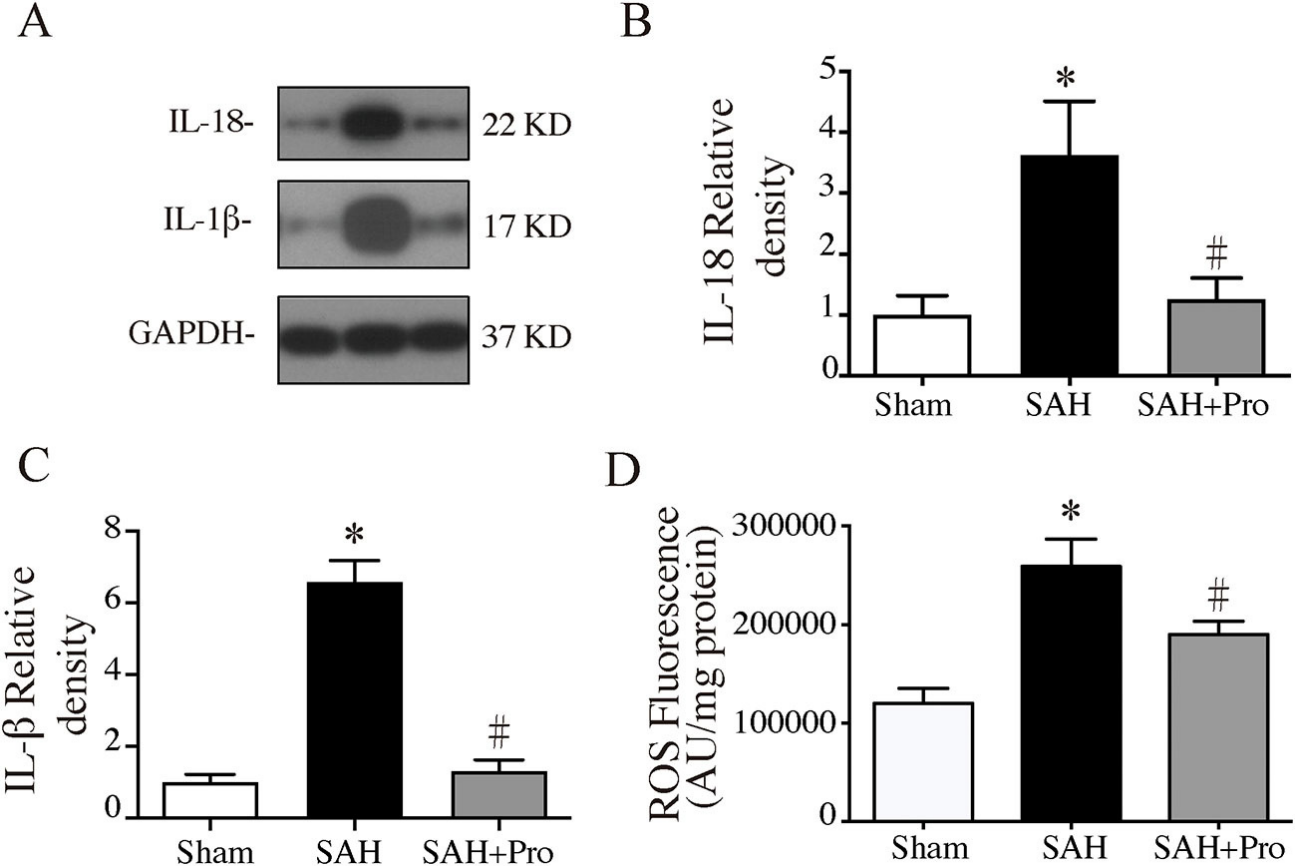
**(A)** Representative western blotting images of interleukin-18 (IL-18) and interleukin-1β (IL-1β). **(B)** IL-18 protein levels in sham, SAH, and SAH + pro groups, *n* = 6 each group. *p < 0.05 vs. sham group, #*p* < 0.05 vs. SAH group. **(C)** IL-1β protein levels in sham, SAH, and SAH + pro group, *n* = 6 each group. *p < 0.05 vs. sham group, #*p* < 0.05 vs. SAH group. **(D)** The quantification of ROS levels in the ipsilateral basal cortex at 24 h after SAH, *n* = 6 each group. *p < 0.05 vs. sham group, #*p* < 0.05 vs. SAH group.

The activation of inflammasome leads to the processing and secretion of the pro-inflammatory cytokines IL-1β and IL-18. To determine the expressions of IL-1β and IL-18, western blot was performed. The expressions of IL-1β and IL-18 were significantly increased 24 h after SAH, whereas the probenecid treatment markedly decreased the levels of IL-1β and IL-18 in SAH rats (*p* < 0.05, Figures 4A–C). The pro-inflammatory cytokines increased mitochondrial-generated ROS production, which indicated the cell damage after SAH. ROS levels were significantly higher in the SAH group than that in the sham group, and probenecid treatment markedly decreased the ROS content compared with SAH rats (*p* < 0.05, Figure 4D). These results suggested that treatment with probenecid inhibited the AIM2 inflammasome activation and resulted in less pro-inflammatory cytokines and ROS generation after SAH induction.

### Probenecid Inhibited Hemin-Induced AIM2 Inflammasome Activation and Neuronal Death *in vitro*

**Figure 5:**
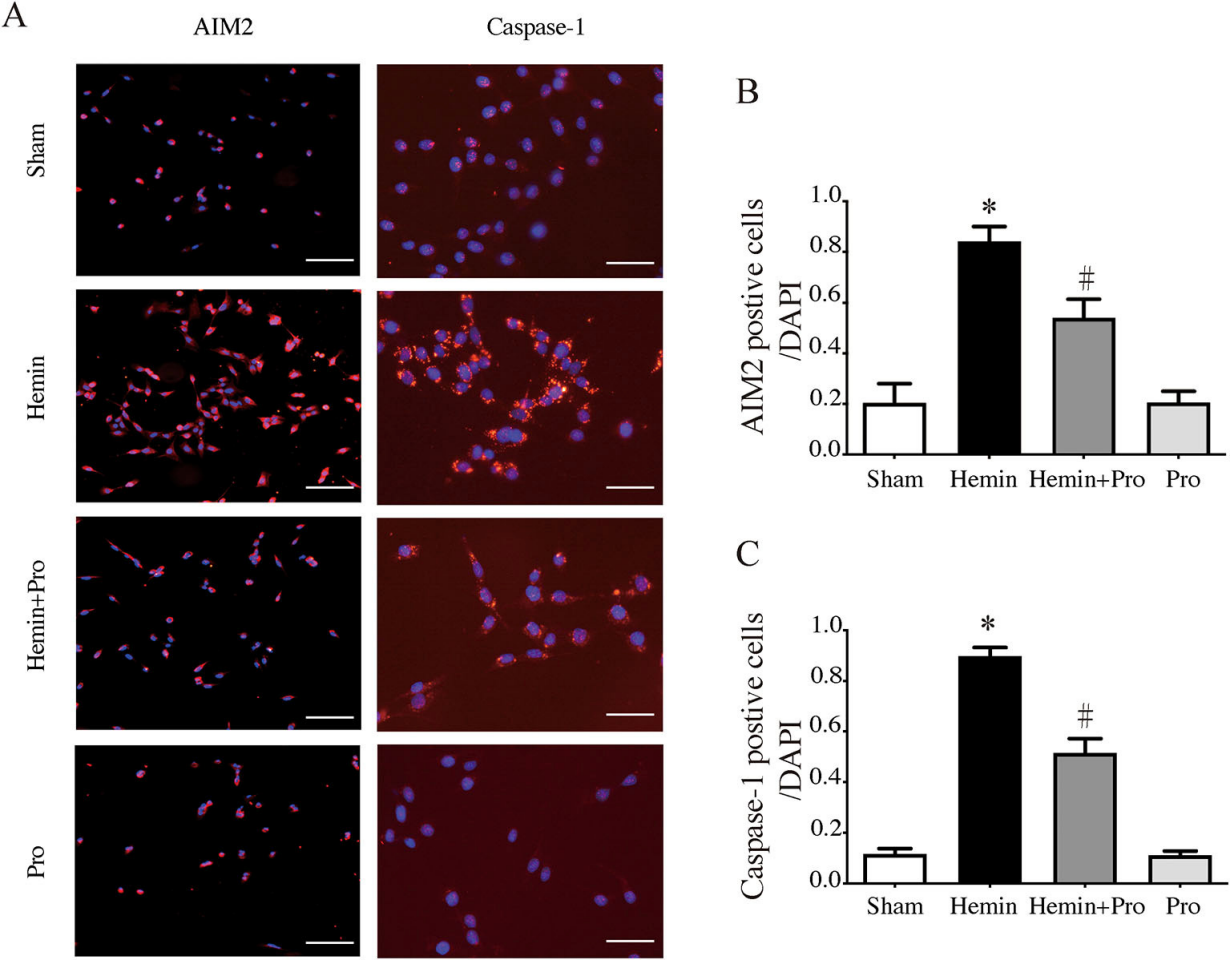
**(A)** Representative micrographs showing immunofluorescence labeling of AIM2 and caspase-1 in cultured HT22 cells of sham, hemin, hemin + pro, and pro groups. Left scale bar =100 μm, right scale bar =50 μm. **(B)** The proportion of AIM2-positive cells in sham, hemin, hemin + pro, and pro groups. *p < 0.05 vs. sham group, #*p* < 0.05 vs. hemin group. **(C)** The proportion of caspase-1-positive cells in sham, hemin, hemin + pro, and pro group. *p < 0.05 vs. sham group, #*p* < 0.05 vs. hemin group.

**Figure 6:**
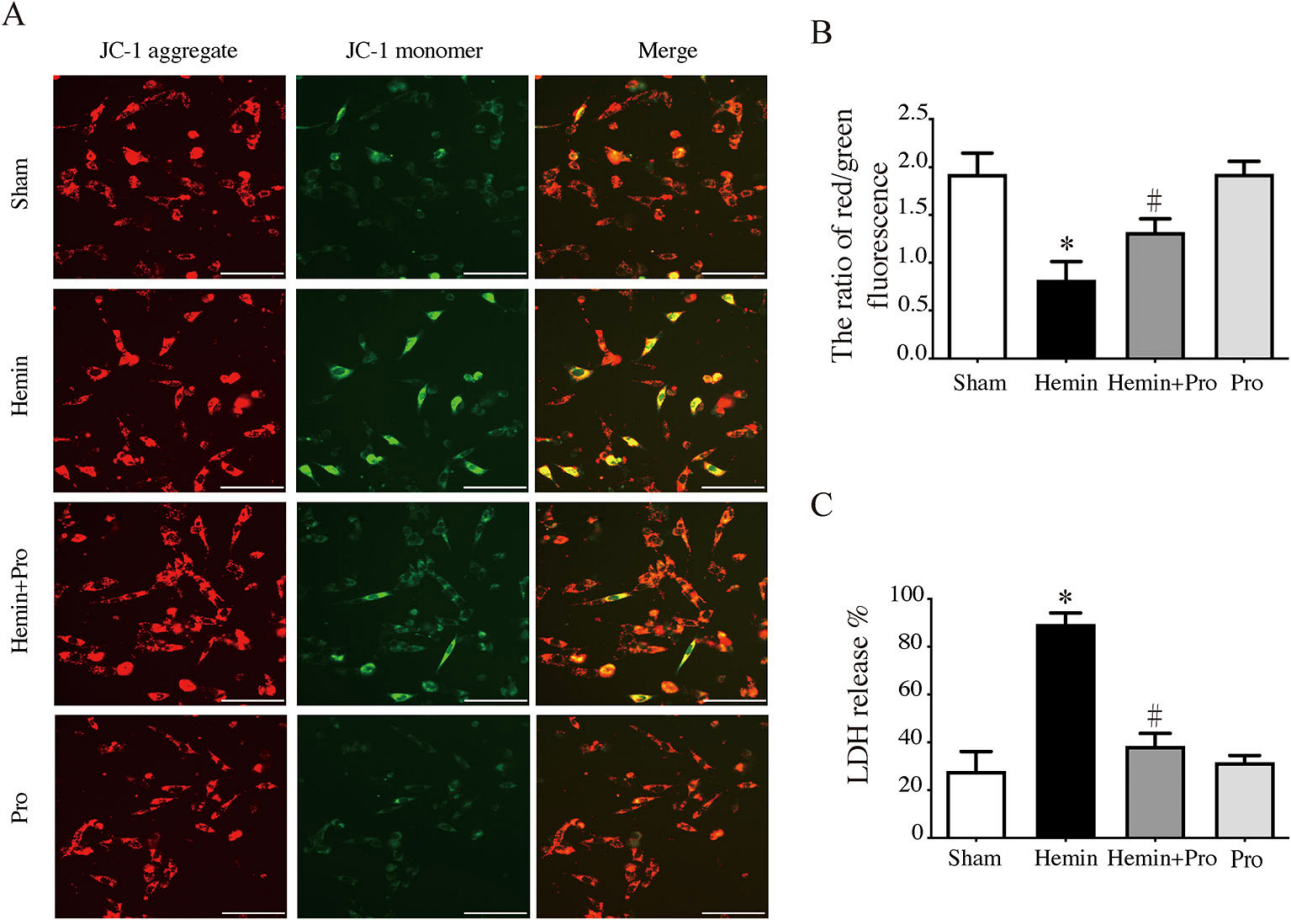
**(A)** Representative micrographs show JC-1 staining in cultured HT22 cells of sham, hemin, hemin + pro, and pro group. Scale bar = 50 μm. **(B)** The ratio of red–green fluorescence in JC-1 staining of sham, hemin, hemin + pro, and pro group. *p < 0.05 vs. sham group, #*p* < 0.05 vs. hemin group. **(C)** The release proportion of media lactate dehydrogenase in sham, hemin, hemin + pro, and pro group. *p < 0.05 vs. sham group, #*p* < 0.05 vs. hemin group.

AIM2 inflammasome was primarily found in neurons. To investigate whether AIM2-mediated neuronal cell injury after SAH, we treated HT22 cells with hemin to mimic SAH injury for neurons. Immunostaining showed that AIM2-positive and ASC-positive cells were significantly elevated after hemin stimulation. The treatment of probenecid reduced the proportion of positive cells (*p* < 0.05, Figure 5). JC-1 staining was used to detect mitochondrial membrane potential, which was associated with ROS generation and cell damage. Normal membrane potential indicated red fluorescence intensity in the control group. Treatment of 75 μm hemin increased green fluorescence intensity, which represented the decline of membrane potential in HT22 cells. Co-incubation with probenecid attenuated hemin-induced collapse of membrane potential (Figure 6A). Quantification of JC-1 fluorescence intensity (red–green florescent area) is shown in Figure 6B. Moreover, the effect of probenecid treatment on protecting HT22 cells from hemin-induced cell injury was evaluated by LDH assay. Media LDH level was significantly increased after treating HT22 cells with 75 μm of hemin compared with the control group (*p* < 0.05, Figure 6C). This was attenuated by the treatment of probenecid (*p* < 0.05, Figure 6C). These results indicated that probenecid treatment alleviated hemin-induced AIM2 inflammasome activation and reduced neuronal cell death *in vitro*.

## Discussion

The results obtained in this study demonstrated that AIM2 inflammasome was activated in the acute stage of SAH, along with the expression of the pannexin-1 channel. The AIM2 inflammasome activation is related to the neuronal cell death after SAH. Treatment with specific pharmacological pannexin-1 channel inhibitor probenecid could attenuate AIM2 inflammasome activation, consequently reduce EBI, and improve neurological dysfunction. Further *in vitro* study showed that probenecid inhibited hemin-induced neuronal AIM2 inflammasome activation and alleviated mitochondrial dysfunction and neuronal cell death. Take a whole, this study presented evidence for the neuroprotection mechanism of treatment with pannexin-1 inhibitor probenecid on AIM2 inflammasome activation-induced brain injury after SAH.

Several studies have shown that EBI is an important factor, which leads to the poor prognosis of SAH, which is involved in many pathological processes (2). Neuroinflammation contributes to the pathogenesis of post-SAH EBI with the activation of inflammatory cells and the increase of pro-inflammatory cytokines (26–28). Extensive studies have shown that the increased pro-inflammatory cytokines, which include IL-18, IL-1β, TNF-α, and IL-6, were closely associated with EBI and neurological dysfunction after SAH (26, 28). Among the pro-inflammatory cytokines, IL-1β is considered a key mediator of the neuroinflammatory response after SAH. The secretion and maturation of IL-1β contribute to the MMP-9-induced blood–brain barrier (BBB) disruption, subsequent brain edema, and neuronal cell death following SAH (29). A previous study demonstrated that the selective inhibitor of IL-1β blocked JNK-mediated MMP-9 activation and reduced BBB disruption in SAH rats (29). The production and activity of IL-1β depend on the activation of caspase-1 by the inflammasome. Inflammasomes are cytosolic multiprotein complexes that typically have three main components: a cytosolic pattern-recognition receptor, the enzyme caspase-1, and an adaptors (30). The activation of caspase-1 cleaved the inactive pro-IL-1β and pro-IL-18 into mature cytokines and induce a pro-inflammatory cell death, pyroptosis (5, 31). An increasing number of evidence has shown that Z-VAD-FMK or AC-YVAD-CMK, caspase-1 inhibitors, could reduce inflammasome activation-mediated inflammatory response and neuronal cell death after stroke (32–34).

The way to classify inflammasome complexes is based on the receptor that initiates signaling. Inflammasome signaling in the CNS is mainly attributed to microglia, and NLRP3 is currently the most widely studied inflammasome in the acute phase of SAH. Inhibition of microglial inflammasome activation has been shown to attenuate EBI after SAH (34, 35). Recent studies have demonstrated that inflammasome components are also expressed in other CNS cell types, which include neurons, astrocytes, oligodendrocytes, and endothelial cells, which served as important functions as microglial inflammasomes (30, 31). AIM2 inflammasome is a highly specific inflammasome mainly found in neurons and can be activated by dsDNA. Once activated, AIM2 recruits ASC to cleave pro-caspase-1, which leads to the maturation of IL-1β and IL-18 and neuronal pyroptosis. AIM2 inflammasome-mediated neuroinflammation and pyroptotic neuronal cell death have been validated in some CNS diseases (36, 37). A study found that AIM2 inflammasomes contributed to ASC to acute brain injury independent of NLRP3 after stroke (36). Further investigation demonstrated thatAIM2 inflammasome activation contributed to EBI after SAH through the GSDMD-induced pyroptosis (10). Our study presented the time course of AIM2 inflammasome activation and AIM2 inflammasome-mediated neuronal cell death in the acute stage of SAH.

As a dsDNA sensor, AIM2 could respond to a variety of cytosolic DNA, which includes viral DNA and bacterial DNA, from pathogens and hosts. The levels of nuclear and mitochondrial DNA in the CSF of patients with SAH were found to be significantly elevated and sequentially activated AIM2 inflammasome (10). The dsDNA fragmentation in the CSF after SAH is mainly released by disintegrating and dying cells, whose key feature is plasma membrane pore formation (38). Once activated by dsDNA, AIM2 inflammasome activation-mediated neuronal pyroptosis could perpetuate dsDNA fragmentation releasing in a positive feedback loop (7). Pannexin-1 is ubiquitously expressed in various cell types in the brain, which include neurons, microglia, astrocytes, and endothelial cells (39, 40). As a channel with a broad range of permselectivity, pannexin-1 participates in large pore formation on the cell membrane with an ATP-dependent pathway (11). Pannexin-1 mediates the secretion and uptake of multiple solutes and is associated with calcium signaling *via* ATP release and the activation of the P2X7 receptor (12). The evidence has shown that the pannexin-1 channel was an important mediator of neuroinflammation, and the inhibition of pannexin-1 could reduce NLRP3 inflammasome activation after stroke (41, 42). The microscopic manifestations of post-SAH brain injury are various types of cell damage and death, accompanied by the release of fragmented dsDNA and ROS. Large pore formation mediated by pannexin-1 allowed dsDNA and ROS to enter cells, thereby activating neuronal and glial inflammasome. Further studies have confirmed that the pannexin-1 channel activated the inflammasome in neurons and astrocytes (16). Probenecid, as a specific pannexin-1 inhibitor, has been proven to protect against ischemic stroke by reducing inflammation and brain edema (22). In this study, we want to investigate the association between the pannexin-1 channel and AIM2 inflammasome activation after SAH and whether pharmacological pannexin-1 inhibitor probenecid provides neuroprotective effects on EBI following SAH. We found that both AIM2 and pannexin-1 levels increased with a marked accumulation at 24 h after SAH. Our *in vitro* experiment suggested the protective effect of probenecid on hemin-mediated neuronal cell damage, at least partly based on the inhibition of neuronal AIM2 inflammasome activation. However, it is not determined whether the neuroprotective effect of probenecid *in vivo* is directly based on the inhibition of neuronal AIM2 inflammasome or indirectly through other glial cell acting on neuronal inflammasome changes. More studies are needed to explore the effect of probenecid in the interaction of neuronal and glial inflammasome.

In this study, we investigated the protective effect of pannexin-1 inhibitor probenecid on EBI after SAH and provided the potential mechanisms lying behind it. However, there are several limitations to this study. As a drug that has been used in the clinic, the pharmacokinetic processes of probenecid are known, but the dose–effect relationship should be investigated in this study. The specific activation mechanism between the pannexin-1 channel and AIM2 inflammasome still needs further research. The dsDNA level should be measured after the SAH at different times and in different groups. The glial and other neuronal inflammasome-mediated neuroinflammation and pyroptotic neuronal cell death should be taken into consideration in the animal experiment. The studies on the anti-inflammation effect of probenecid are also in great demand in the hemin-mediated cell culture model. The severity of post-SAH brain injury might be associated with age and gender in animal and clinical experiments (43–45), which were not considered in this study. We plan to apply our findings in future articles for aged and female groups.

In conclusion, this study demonstrated the neuroprotective effect of probenecid on EBI after SAH and explored the underlying mechanisms. It showed that the expression of the pannexin-1 channel was increased and neuronal AIM2 inflammasome was activated following SAH. As a pannxin-1 inhibitor, probenecid treatment could reduce AIM2 inflammasome activation and pro-inflammatory cytokines secretion, alleviate mitochondrial damage, alleviate attenuate brain edema, and improve neurological dysfunction following SAH.

## Data Availability Statement

The original contributions presented in the study are included in the article/Supplementary Material, further inquiries can be directed to the corresponding authors.

## Ethics Statement

The animal study was reviewed and approved by Zhejiang University.

## Author Contributions

YZ: methodology and writing—original draft. WT: methodology and writing—original draft. HZ: conceptualization. YP: methodology. XY: writing—review and editing. FY: conceptualization, writing—review and editing, and project administration. SC: conceptualization, writing—review and editing, project administration, and funding acquisition. All authors contributed to the article and approved the submitted version.

## Funding

This study was supported by grant 81801144 from the National Natural Science Foundation of China.

## Conflict of Interest

The authors declare that the research was conducted in the absence of any commercial or financial relationships that could be construed as a potential conflict of interest.

## Publisher's Note

All claims expressed in this article are solely those of the authors and do not necessarily represent those of their affiliated organizations, or those of the publisher, the editors and the reviewers. Any product that may be evaluated in this article, or claim that may be made by its manufacturer, is not guaranteed or endorsed by the publisher.
